# Multisystemic Sarcoidosis in the Primary Care Setting: A Case Report

**DOI:** 10.7759/cureus.75490

**Published:** 2024-12-10

**Authors:** Ana Matos, Marta Bordalo, Hugo Silva

**Affiliations:** 1 Family Medicine, Unidade de Saúde Familiar (USF) Amato Lusitano, Unidade Local de Saúde (ULS) de Amadora/Sintra, Amadora, PRT

**Keywords:** case report, cutaneous sarcoidosis, extrapulmonary manifestations, lymphadenopathy, multisystemic sarcoidosis, parotid swelling, primary care, sarcoidosis

## Abstract

Sarcoidosis is a rare, multisystemic disease of unknown etiology, characterized by noncaseating granulomas in various organs. The disease often presents with nonspecific symptoms that complicate the diagnosis.

We describe the case of a 31-year-old woman who presented to her family doctor with weight loss, cervical lymphadenopathy, parotid edema, and cutaneous lesions, initially raising suspicion of a lymphoproliferative disorder. A thoraco-abdominopelvic CT scan revealed extensive lymphadenopathy and multiorgan involvement, consistent with sarcoidosis. While awaiting outpatient evaluation by internal medicine, the patient developed arthralgias and was subsequently admitted for further investigation. She was ultimately diagnosed with sarcoidosis with multiorgan involvement, including the lymphatic, pulmonary, lacrimal, salivary, hepatic, splenic, and cutaneous systems. Retrospective analysis suggested that parotitis during pregnancy five years earlier was likely the initial manifestation of the disease.

This case underscores the importance of early recognition and multidisciplinary management in sarcoidosis, particularly when the disease has widespread systemic involvement and nonspecific symptoms. Family physicians, often the first point of contact, play a crucial role in identifying early signs, ensuring appropriate referrals, and coordinating long-term follow-up.

## Introduction

Sarcoidosis is a multisystemic disease of unknown etiology, characterized by the formation of noncaseating granulomas in several organs. Its diagnosis is challenging owing to its rarity, lack of sensitive and specific diagnostic tests, and heterogeneous presentation [1]. 

Intrathoracic involvement occurs in approximately 90% of patients, with the most common findings being symmetric bilateral perihilar lymphadenopathy and diffuse pulmonary nodules in the upper lobes [1]. Extrapulmonary manifestations occur in 30%-50% of cases [2]. Cutaneous involvement is common but often underdiagnosed due to variability in lesion morphology. Salivary gland involvement is an uncommon manifestation, observed only in approximately 4%-6% of cases, and frequently presents as parotid edema [1,3].

The diagnosis of sarcoidosis relies on three main criteria: a compatible clinical and/or radiological presentation, histological evidence of noncaseating granulomatous inflammation, and the exclusion of other granulomatous diseases. Differential diagnoses include malignancies such as Hodgkin’s and non-Hodgkin lymphomas, infectious etiologies (e.g., mycobacterial, bacterial, and fungal infections), pneumoconiosis, hypersensitivity pneumonitis, autoimmune disorders, primary immunodeficiencies, and drug toxicity [4]. 

The clinical course of sarcoidosis is variable. While many patients experience spontaneous resolution, approximately 20% develop permanent symptoms, typically due to pulmonary fibrosis. Mortality rates range from 6% to 7%, primarily attributed to pulmonary or cardiac complications [4]. Corticosteroids are the first-line treatment, followed by immunosuppressants and cytotoxic agents. Antitumor necrosis factor (anti-TNF) agents are reserved for severe and/or refractory cases [5,6].

We describe the case of a 31-year-old woman who presented to her family doctor with fatigue, weight loss, lymphadenopathy, parotid swelling, and skin lesions. Initial suspicion focused on lymphoproliferative disease, but the thoraco-abdominopelvic CT scan revealed findings indicative of sarcoidosis. As further tests were required, she was referred to the internal medicine department, where sarcoidosis with extensive extrapulmonary involvement was confirmed.

## Case presentation

A 31-year-old woman presented to her family doctor at the Family Care Unit (FCU). She had previously worked as a secretary but was unemployed at the time of the consultation. She reported no environmental or occupational risk factors. Her vaccination record was up-to-date, and she had been under regular care at the FCU since 2017. The patient reported previous tobacco use but no alcohol or drug use. Her medical history included congenital right-sided peripheral facial nerve palsy and right-sided parotitis of an uncertain etiology in 2019. She was taking a combined oral contraceptive and had no known drug allergies.

**Figure 1:**
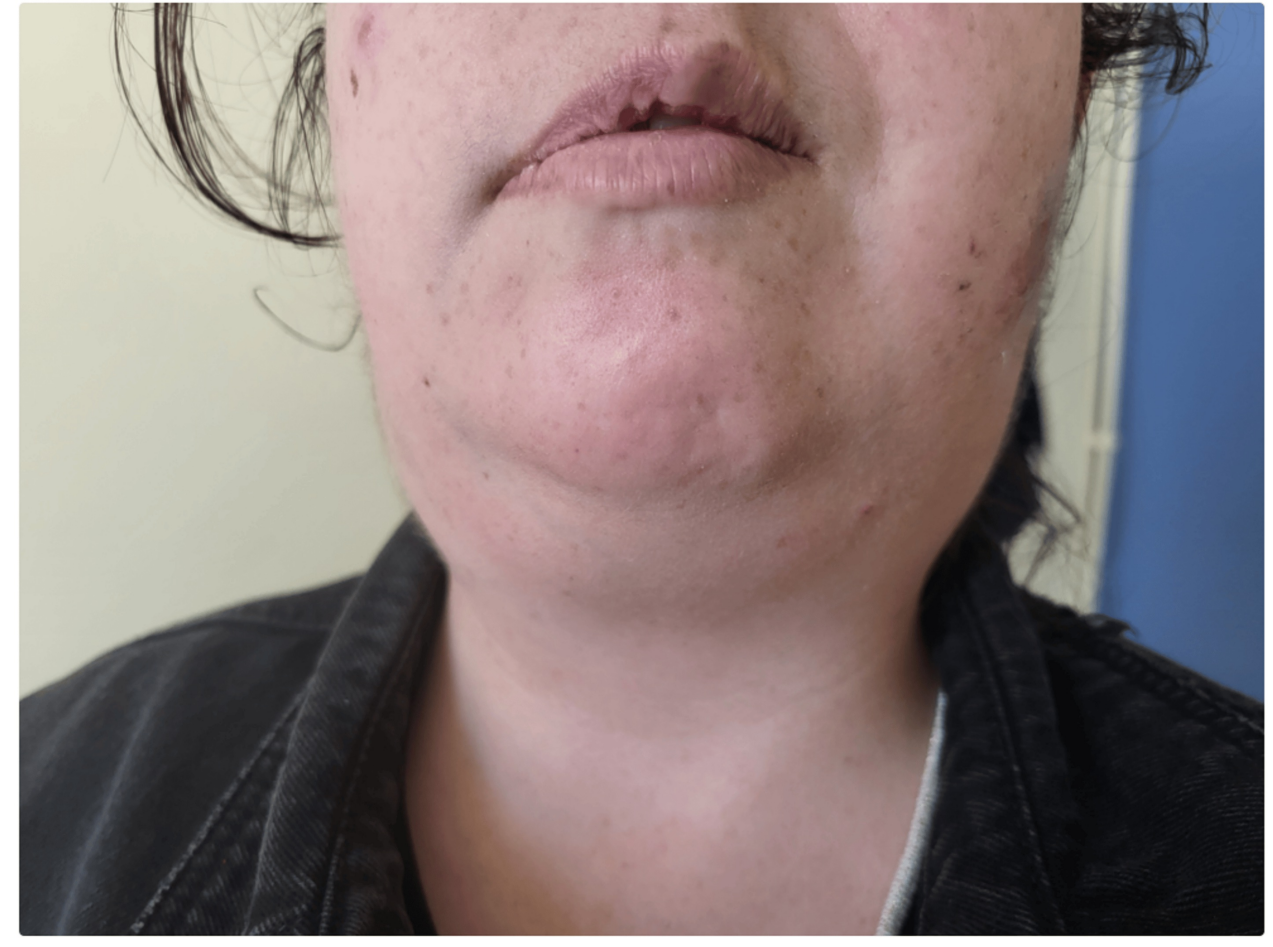
Right-sided peripheral facial nerve palsy and parotid and cervical edema. The patient's photograph was taken with permission.

**Figure 2:**
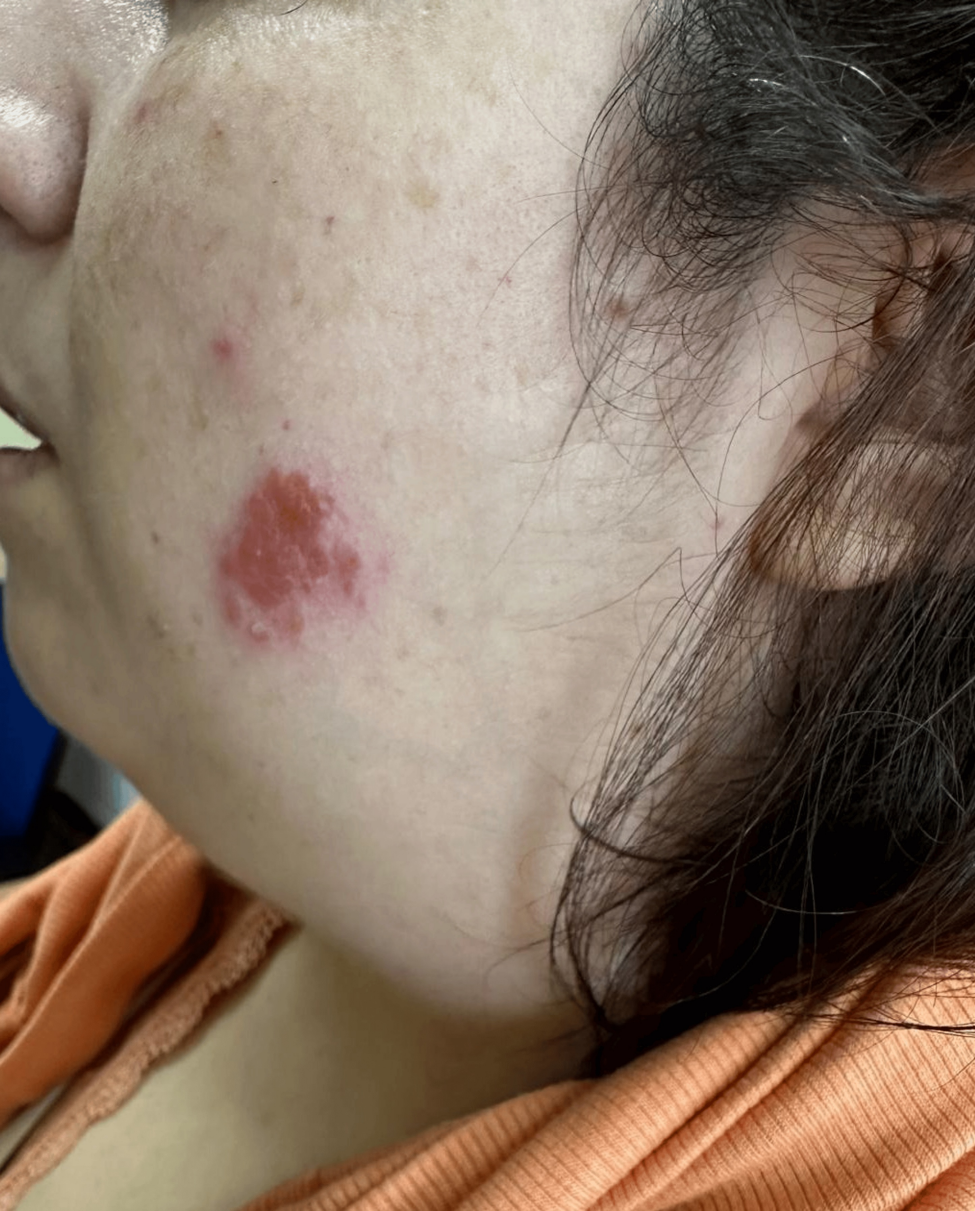
Lesion on the left cheek (20 mm x 20 mm). The patient's photograph was taken with permission.

**Figure 3:**
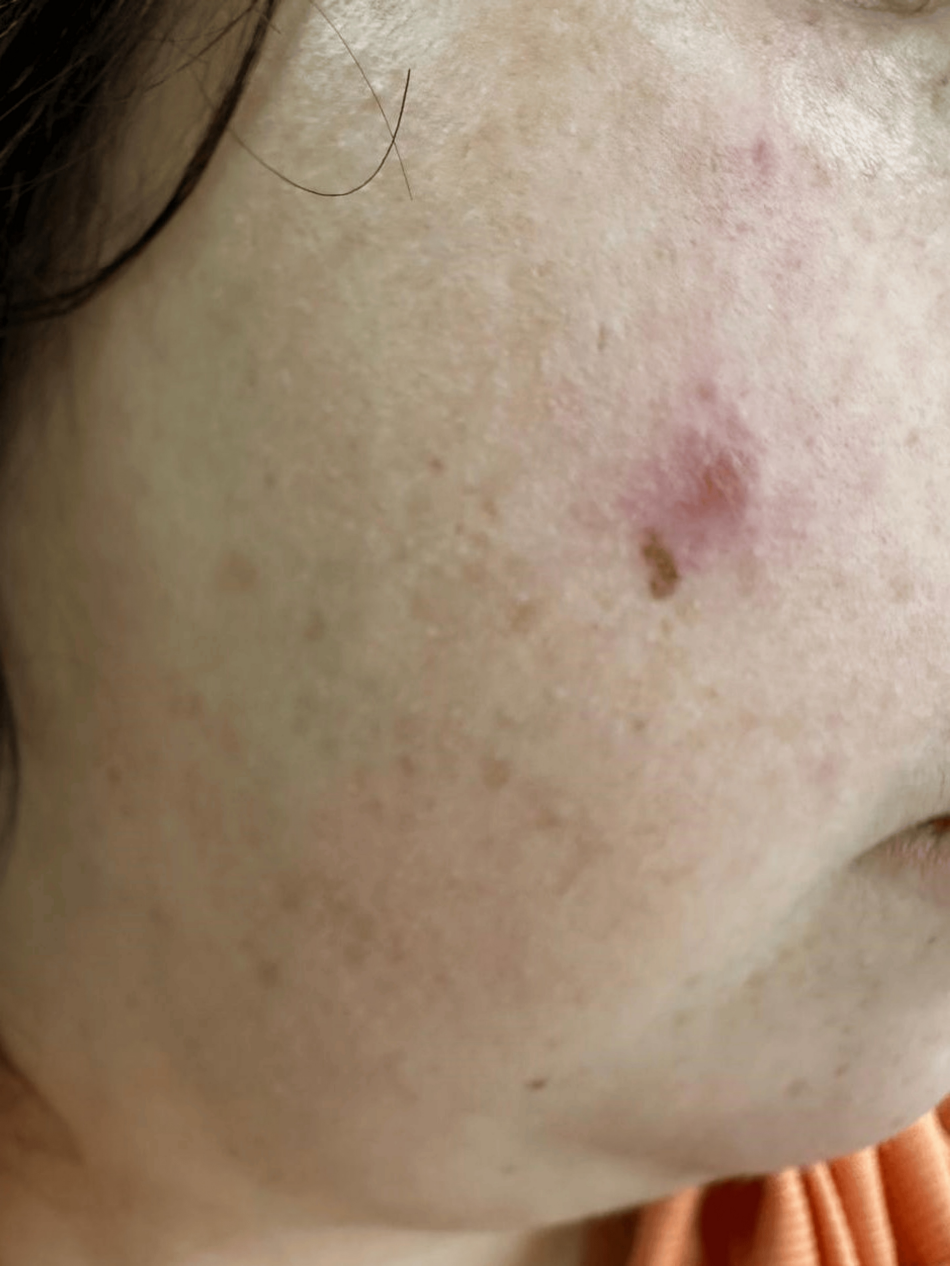
Lesion on the right cheek (10 mm x 10 mm). The patient's photograph was taken with permission.

**Figure 4:**
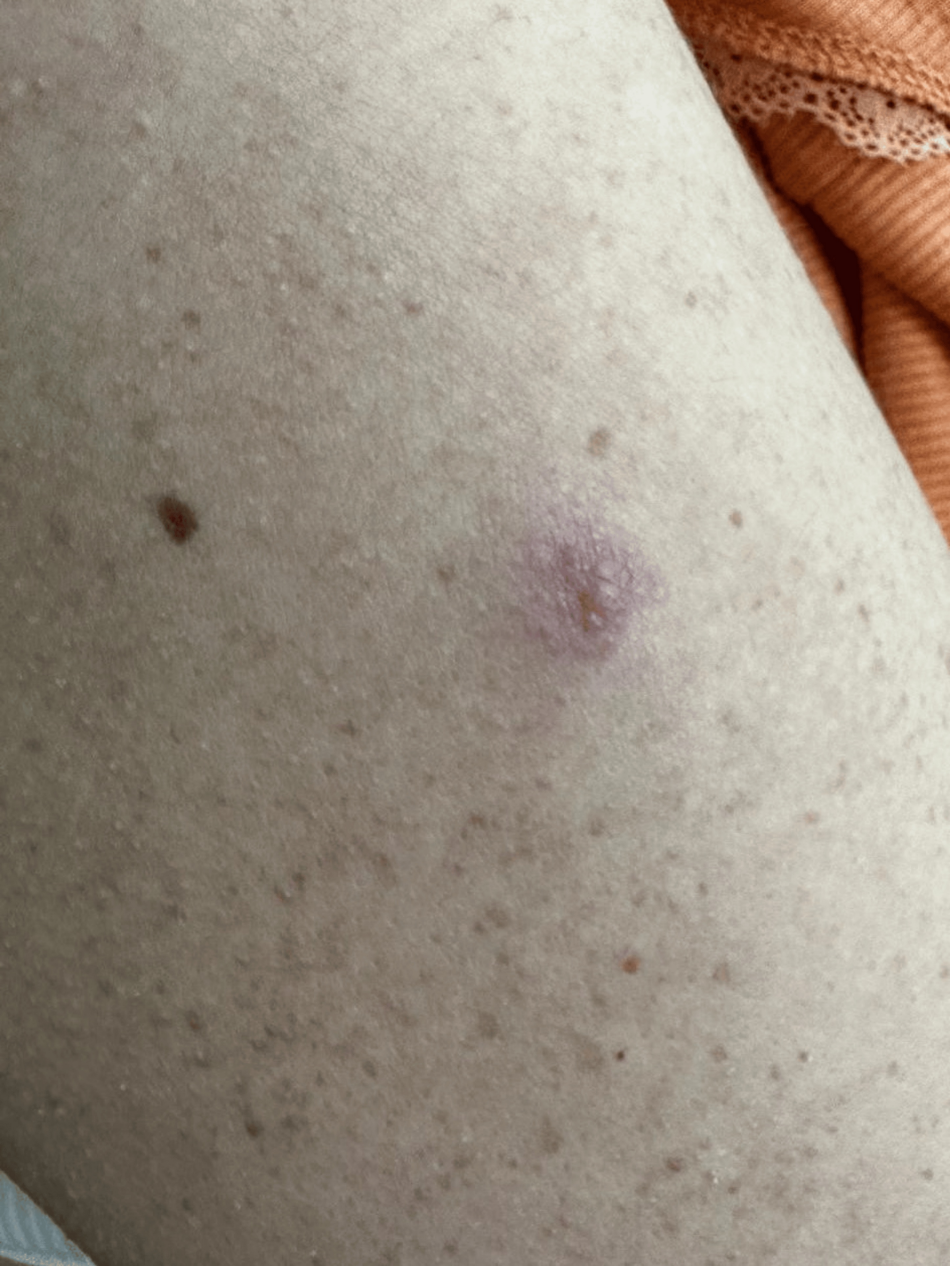
Cutaneous lesion on the left upper limb (5 mm x 5 mm). The patient's photograph was taken with permission.

The patient complained of fatigue and 10-kg weight loss over the past year. She denied any fever, night sweats, cough, or dyspnea. Physical examination revealed right-sided facial nerve palsy and bilateral parotid gland edema, particularly on the right side. Cervical palpation detected a firm, tender mass on the right side, approximately 20 mm × 20 mm in size (Figure 1). The patient also had infiltrated firm cutaneous plaques and papules on the face, back, and upper limbs, which were not painful or pruritic. The largest lesions were on the left cheek (20 mm × 20 mm) (Figure 2) and right cheek (10 mm × 10 mm) (Figure 3). Additional lesions were found on the left upper limb (two lesions) and the back (three lesions), each measuring approximately 5 mm × 5 mm (Figure 4). According to the patient, the cutaneous lesions had been present for over a year. She also reported noticing facial and cervical asymmetry for several years, which she had attributed to the sequelae of congenital facial nerve palsy and parotitis. In light of these findings, a cervical ultrasound and blood tests were ordered.

Four weeks later, the patient presented with blood test results showing a total white blood cell count of 3.5 × 10³/μL (normal range, 4.0-10.0 × 10³/μL), 18.2% lymphocytes (normal range, 20.0%-40.0%), and a platelet count of 144 × 10³/μL (normal range, 150-400 × 10³/μL). The erythrocyte sedimentation rate (ESR) was 20.0 mm/hour (normal range, <20.0 mm/hour), and the C-reactive protein (CRP) was within normal limits at 0.53 mg/dL (normal range, 0.05-1.00 mg/dL). Liver and renal function tests were normal, and serological tests for Epstein-Barr virus (EBV) and HIV were negative. Cervical ultrasonography revealed bilateral adenopathy conglomerates involving all levels of the cervical lymphatic chains. Enlarged intraparotid and submandibular lymph nodes, several measuring approximately 20 mm, were also observed, exhibiting vascularity. 

**Figure 5:**
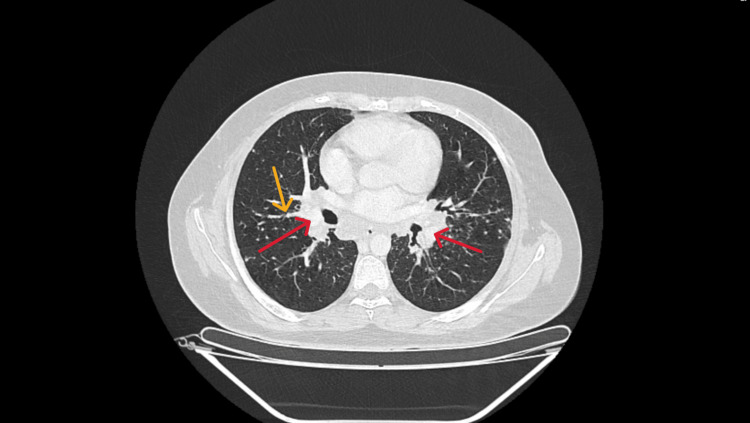
Chest CT scan. Chest CT imaging showing bilateral hilar lymphadenopathy (red arrows) and micronodularity in the lung parenchyma with a perilymphatic distribution (yellow arrow).

**Figure 6:**
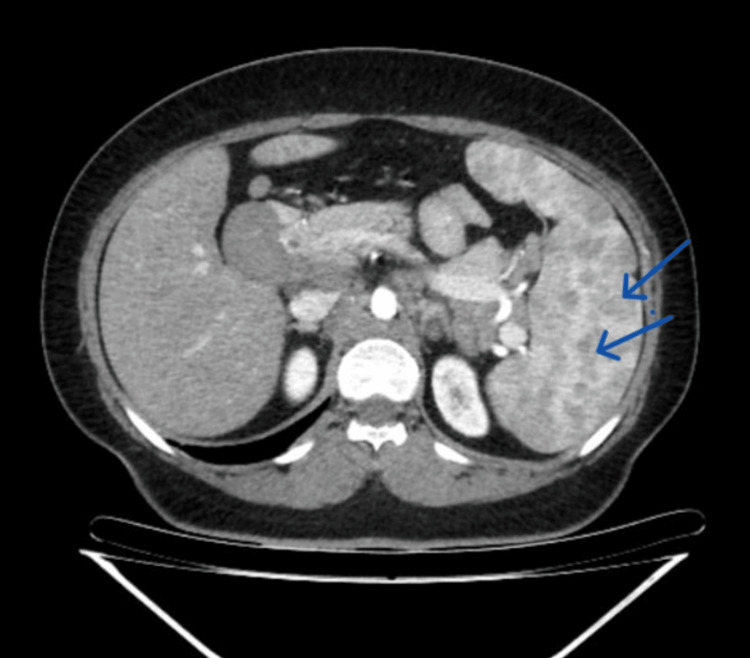
Abdominal CT scan. Abdominal CT imaging revealing splenomegaly with nodular confluent lesions (blue arrows).

These findings, along with the patient's clinical presentation, raised concern for lymphoproliferative disorder. A thoraco-abdominopelvic CT scan was subsequently performed to assess for systemic lymphadenopathy and organ involvement. The scan revealed dispersed pulmonary micronodularity with a perilymphatic distribution, primarily in the upper lobes (Figure 5). Multiple calcified lymph nodes were observed. Bilateral axillary adenopathy was present, with the largest node measuring 29 mm × 14 mm, along with left internal mammary, diaphragmatic, mediastinal, and hilar involvement, including a subcarinal conglomerate measuring 42 mm × 27 mm. The celiac and hepatic nodes were affected, with the largest measuring 36 mm × 18 mm. Additionally, lymph node involvement was observed in the retrocrural, peripancreatic, splenic, mesenteric, and predominantly lumbar-aortic regions, with the largest measuring 36 mm × 17 mm. The right and left primitive and external iliac lymph nodes measured 50 mm × 22 mm and 48 mm × 18 mm, respectively, with bilateral inguinal lymphadenopathy, the largest node measuring 17 mm × 13 mm. The liver was slightly enlarged, with multiple millimetric and hypointense lesions. The spleen was moderately enlarged, measuring approximately 15 cm along its longitudinal axis, with multiple confluent and hypodense centimetric lesions (Figure 6). Nonobstructive renal calculi were observed, two of which were located in the right lumbar ureter, with the largest measuring approximately 10 mm, resulting in mild upstream hydronephrosis.

The clinical presentation, along with the CT findings, raised the possibility of sarcoidosis as a potential diagnosis. 

Given the differential diagnosis of a lymphoproliferative disorder and sarcoidosis, and in the absence of additional laboratory and histopathological tests available in primary care, the patient was referred to the outpatient internal medicine department. While awaiting evaluation, she presented to the FCU with a new-onset severe inflammatory arthralgia affecting the hips, interphalangeal joints, and elbows. A review of the hospital referral platform revealed that the internal medicine appointment had not yet been scheduled. Due to the development of new symptoms and the unclear etiology of her systemic manifestations, the decision was made to refer the patient to the emergency department, along with a detailed summary of the investigations conducted thus far.

An internal medicine team assessed the patient in the emergency department and admitted her for a comprehensive evaluation. Throughout the hospitalization period, the patient remained hemodynamically stable. Admission laboratory results revealed lymphopenia (0.53 × 10^3^/µL), thrombocytopenia (113 x 10^3^/µL), and elevated CRP (1.18 mg/dL) and ESR (62 mm/hour). Additionally, her serum angiotensin-converting enzyme (ACE) level was elevated at 423 U/L (Table 1).

**Table 1:** Analytical results on hospital admission.

Test result		Reference values
Hemoglobin	12.4	12.0-15.3 (g/dL)
Mean corpuscular volume	88.6	80.0-97.0 (fL)
White blood cells	3.0	4.0-11.0 (x 10^3^/µL)
Neutrophils	1.80	1.90-7.50 (x 10^3^/µL)
Eosinophils	0.17	0.00-0.50 (x 10^3^/µL)
Basophils	0.02	0.00-0.20 (x 10^3^/µL)
Lymphocytes	0.53	1.00-4.80 (x 10^3^/µL)
Monocytes	0.48	0.10-1.00 (x 10^3^/µL)
Platelets	113	150-450 (x 10^3^/µL)
C-reactive protein	1.2	<0.5 (mg/dL)
Erythrocyte sedimentation rate	62	<12 (mm/hour)
Urea	31	16-39 (mg/dL)
Creatinine	0.71	0.50-0.90 (mg/dL)
Sodium	139	135-145 (mEq/L)
Potassium	3.9	3.5-5.1 (mEq/L)
Calcium	10.5	8.6-10.2 mg/dL)
Total bilirubin	0.3	<1.2 (mg/dL)
Direct bilirubin	0.1	<0.2 (mg/dL)
Aspartate aminotransferase	25	0-32 (U/L)
Alanine aminotransferase	23	0-33 (U/L)
Gamma-glutamyl transferase	71	0-40 (U/L)
Alkaline phosphatase	106	35-105 (U/L)
Lactate dehydrogenase	208	100-250 (U/L)
Angiotensin-converting enzyme	423	8-52 (U/L)

The interferon-gamma release assay (IGRA) was negative. Serological tests for hepatitis virus, HIV, EBV, *Leishmania *spp., *Coxiella burnetii,* *Rickettsia conorii,* *Treponema pallidum*, *Borrelia burgdorferi, Bartonella henselae*, *Cytomegalovirus*, and parvovirus were all negative. Electrocardiography (ECG) revealed no abnormalities. Gallium-67 scintigraphy indicated uptake in previously noted areas, displaying the panda sign, characterized by symmetrical uptake in the lacrimal, parotid, and salivary glands. An excisional biopsy of the inguinal lymph node revealed granulomatous lymphadenitis. The patient was evaluated by the dermatology department, and biopsies of the facial and upper limb lesions were consistent with sarcoidosis.

These findings established a diagnosis of sarcoidosis, characterized by extensive systemic involvement, including the lymphatic, salivary and lacrimal gland, pulmonary, hepatic, splenic, and cutaneous systems. Ophthalmological assessments ruled out ocular complications, while an outpatient urology appointment for nephrolithiasis evaluation was pending at the time of discharge.

The patient was discharged on the 10th day of hospitalization, with a prescription for methotrexate 10 mg weekly, folic acid 5 mg weekly, hydroxychloroquine 400 mg daily, prednisolone 40 mg daily, and topical tacrolimus 1 mg/g daily. 

She returned for a follow-up consultation at the FCU one month after discharge, showing a reduction in cervical swelling, improvement in the cutaneous lesions, and resolution of articular pain. 

Following the diagnosis of sarcoidosis, a retrospective review of the patient's clinical records revealed that five years earlier, at 28 weeks of gestation, she had presented to the emergency department with right facial edema and a 6-kg weight loss since the onset of her pregnancy. Cervical ultrasound indicated heterogeneity and thickening of the right parotid gland, along with reactive lymph nodes. The patient was prescribed a 10-day course of amoxicillin and clavulanic acid. Although she reported slight improvement, she indicated that the edema had persisted since then.

## Discussion

This clinical case illustrates a presentation of sarcoidosis with extensive systemic involvement, underscoring the pivotal role of primary care doctors in the detection of this condition. The family physician's responsibilities included identifying clinical signs, performing initial diagnostic tests, and providing emotional support throughout the often uncertain diagnostic process.

Patients with sarcoidosis often present with nonspecific symptoms, making primary care the initial point of contact. In this case, the patient presented with fatigue, weight loss, skin lesions, parotid swelling, and a cervical mass. Ultrasound findings revealed salivary gland and cervical lymphadenopathy, initially raising suspicion of a lymphoproliferative disorder. However, initial blood tests showed no significant abnormalities. A CT scan revealed pulmonary micronodularity with a perilymphatic distribution, predominantly in the upper lobes, alongside lymphatic, hepatic, and splenic involvement. These radiological findings, particularly the pulmonary pattern and thoracic lymph node calcifications, were more consistent with sarcoidosis than lymphoma. Additionally, the presence of cutaneous lesions further supported the suspicion of sarcoidosis over lymphoma [2].

Due to the limited diagnostic resources in the primary care setting, the patient was referred for an outpatient hospital visit to obtain a definitive diagnosis. However, given the onset of pain in the context of an uncertain diagnosis, the decision was made to refer the patient to the emergency department for more immediate evaluation and management. Upon admission, the patient's blood tests showed elevated ACE levels, supporting the diagnosis of sarcoidosis. ACE levels are elevated in approximately 75% of patients with untreated sarcoidosis. Although the low sensitivity and specificity limit its diagnostic utility, an ACE level exceeding twice the upper limit of the normal range yields a specificity of over 90% for sarcoidosis [7].

The combination of clinical and radiological findings, along with biopsy results showing noncaseating granulomas and the exclusion of other potential diseases (including malignancy, infections, and occupational-related conditions), led to a final diagnosis of sarcoidosis. Furthermore, gallium-67 scintigraphy revealed the panda sign, characterized by symmetrical uptake in the lacrimal and parotid glands, which further supported the diagnosis of sarcoidosis [6].

Facial peripheral palsy and parotid edema, when accompanied by fever or uveitis, may indicate a rare subtype of sarcoidosis known as Heerfordt-Waldenström syndrome, which occurs in approximately 0.3%-1.3% of patients [1]. However, this diagnosis was ruled out in the current case, as Heerfordt-Waldenström syndrome typically manifests acutely or subacutely, and the patient had a documented history of peripheral facial palsy since birth. Ocular complications were also excluded.

In this case, systemic sarcoidosis possibly evolved over several years. This hypothesis was substantiated by findings indicating that parotid edema was present as early as 2019, likely being the initial manifestation of the disease. This information was only uncovered after diagnosis, highlighting the critical need for a centralized network that provides easy access to clinical records for healthcare professionals. Such a system would facilitate timely information sharing, ultimately improving diagnostic accuracy and patient care.

## Conclusions

This case highlights the complexities of diagnosing sarcoidosis with extensive multisystem involvement. Furthermore, it underscores the need to consider sarcoidosis in the differential diagnosis for patients showing systemic symptoms, generalized lymphadenopathy, and atypical cutaneous presentations.

Primary care, often the first point of contact for patients, plays an essential role in recognizing early symptoms, facilitating referrals, and ensuring long-term follow-up. Family physicians provide essential longitudinal support through symptom management, emotional care, and coordination of ongoing care. This case also underscores the importance of cooperation between primary healthcare and hospital specialties. Such collaboration is crucial for managing complex clinical cases effectively and reducing unnecessary emergency visits.
